# Assessment of adverse events stratified by timing of leadless pacemaker implantation with cardiac implantable electronic devices extraction due to infection: A systematic review and meta‐analysis

**DOI:** 10.1002/joa3.13208

**Published:** 2024-12-26

**Authors:** Naoya Inoue, Yuji Ito, Takahiro Imaizumi, Shuji Morikawa, Toyoaki Murohara

**Affiliations:** ^1^ Department of Cardiology Chutoen General Medical Center Kakegawa, Shizuoka Japan; ^2^ Department of Cardiology Nagoya University Graduate School of Medicine Nagoya Japan; ^3^ Department of General Internal Medicine Chutoen General Medical Center Kakegawa, Shizuoka Japan; ^4^ Department of Advanced Medicine Nagoya University Hospital Nagoya Japan

**Keywords:** all‐cause mortality, cardiac implantable electronic device, leadless pacemaker, reinfection, transvenous lead extraction

## Abstract

**Background:**

Removal of cardiac implantable electronic devices (CIEDs) is strongly recommended for CIED‐related infections, and leadless pacemakers (LPs) are increasingly used for reimplantation. However, the optimal timing and safety of LP implantation after CIED removal for infection remains unclear.

This systematic review and meta‐analysis aimed to assess complication rates (all‐cause mortality and reinfection) when LP implantation was performed simultaneously with or after CIED removal.

**Methods:**

Studies published from 2015 to September 2024 were searched in PubMed, Cochrane Library, and Google Scholar. Observational studies and case series on CIED removal and LP implantation were eligible. The primary outcomes were all‐cause mortality and reinfection post‐LP implantation. Pooled estimates were obtained using the Freedman‐Tukey double arcsine transformation. Study quality was assessed using the MINORS criteria, with data extraction and independent assessment by two authors.

**Results:**

Of 396 records, 16 studies were included in the analysis, with 653 patients (mean age:76.9 years). The incidence of isolated pocket infections was 46.7% (95% CI: 32.7%–61.2%) and systemic infections at 46.3% (95% CI: 29.5%–64.0%). The primary outcome incidence was 19.4% (95% CI: 12.8%–28.3%, *I* ^2^: 0%) for simultaneous CIED extraction and LP implantation compared with 7.79% (4.37%–13.5%, *I* ^2^: 4%) for LP implantation after CIED extraction (*p* = .009). All‐cause mortality rates were 22.8% (95% CI: 15.9%–31.6%, *I* ^2^: 0%) for simultaneous implantation and 8.71% (4.46%–16.3%, *I* ^2^: 21%) after extraction (*p* = 0.008). Reinfection was not observed in any of these studies.

**Conclusion:**

Simultaneous CIED extraction and LP implantation due to infection may be associated with an increased risk of all‐cause mortality.

## Graphical abstract

This meta‐analysis of 16 studies examined the timing of leadless pacemaker implantation during CIED extraction for infections. While simultaneous implantation shows a low risk of reinfection, it is associated with higher all‐cause mortality, and careful consideration of the timing of implantation is recommended.

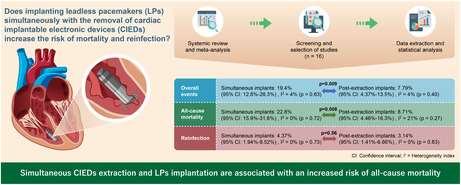

## 1 INTRODUCTION

Cardiac implantable electronic device (CIED) infections are serious complications that affect patient prognosis. The incidence of CIED infection within 12 months post‐implantation ranges from 1.0 to 2.4%.^1^, ^2^, ^3^ Moreover, the infection rate has also increased with increased CIED usage.^4^ In‐hospital or 30‐day mortality rates for CIED infections are reported to be 5%–8%,^5^, ^6^, ^7^ with even higher mortality rates observed in patients who develop CIED‐related infective endocarditis rather than pocket infections.^5^

For appropriate treatment, guidelines strongly recommend the removal of both CIEDs and leads. However, a report using the Nationwide Readmissions Database indicated that transvenous lead extraction (TLE) is performed in only 11.5% of patients with infective endocarditis and CIEDs.^8^ This low rate may reflect the perceived risk of complications associated with TLE, although the actual mortality rate associated with lead extraction is approximately 0.5%.^5^ This suggests a possible evidence‐practice gap.

In patients who are dependent on pacing, there is a need for reimplantation of a new CIED following the removal of the infected device. Recently, leadless pacemakers (LPs) have garnered attention because of their structural features that reduce lead‐related complications (such as lead‐related infections and subclavian vein stenosis) and pocket infections compared to transvenous pacemakers.^9^ Increasing reports have noted LPs selection for reimplantation following CIED infection not only for new implants but also as replacements for infected devices.^10^ Functionally, VDD‐LPs, which are capable of maintaining atrioventricular synchrony,^11^ and dual‐chamber LPs have also demonstrated safety and efficacy, contributing to their adoption.^12^

Arshad et al. reported that among patients who underwent TLE for CIED‐related infective endocarditis, those who received transvenous CIED reimplantation within 14 days of TLE had a higher 1‐year mortality rate (27%) than those who received reimplantation after 14 days (8%); the mortality rate was particularly high (41%) in cases with endocardial vegetations.^13^ Current guidelines by the Japanese Circulation Society and the Japanese Heart Rhythm Society recommend waiting at least 72 h after device removal to confirm negative blood cultures before implanting a new device.^14^ However, many previous reports on CIED extraction and LP implantation due to infection have indicated either no recurrence of infection or a low risk of reinfection.^1^, ^15^ However, these studies often reported mortality during short‐ to mid‐term follow‐up periods, raising concerns that infection may be masked as a competing risk factor. This underscores the need for further statistical evaluations to reassess the safety of this therapeutic strategy.

Therefore, this study conducted a meta‐analysis of the existing research on cases in which CIED removal and LP implantation were performed. Understanding the incidence of LP‐related all‐cause mortality and infection when LP implantation is performed simultaneously with or after CIED removal is critical for determining the optimal LP implantation timing in CIED infection cases.

## 2 METHODS

### 2.1 Data sources and search

Two independent authors (N.I. and Y.I.) conducted a comprehensive search of all studies published between 2015 and September 2024 in PubMed, Cochrane Library, and Google Scholar databases.^16^ The search strategy combined the following search phrases and free terms:

- PubMed search strategy: ([Leadless pacemaker OR leadless cardiac pacemaker] AND [CIED OR lead extraction OR device removal OR TLE OR extraction]) AND (infection OR reinfection OR recurrent infection OR mortality OR death) NOT (Editorial OR Comments)
- Google Scholar search: “Leadless Pacemaker” AND “Extraction” AND “Infection” AND “Concomitant”
- Cochrane Library search: “Leadless Pacemaker” “Extraction” “Infection”

This systematic review was not registered in any public database prior to its completion and no protocol has been published. However, all methodological steps followed standard systematic review practices as outlined by the Preferred Reporting Items for Systematic Reviews and Meta‐Analyses (PRISMA) guidelines (Table S1).^16^ In line with our institutional policy, the systematic review and meta‐analysis did not require approval from the Institutional Review Board.

### 2.2 Study selection

The inclusion criteria were clinical studies and case series with at least two cases reporting CIED removal and LP implantation, and the outcomes of all‐cause mortality or reinfection following LP implantation. The exclusion criteria were as follows.

1. Reviews, case reports, conference abstracts, letters, editorial comments, or books
2. Studies that only reported outcomes related to LP parameters following LP implantation
3. Studies without information on the timing of CIED removal and LP implantation
4. Studies where the indication for CIED removal was non‐infectious (e.g., lead failure, pacemaker malfunction)
5. Studies with an ambiguous or unreported follow‐up period following LP implantation
6. Studies in which devices other than LPs (e.g., S‐ICD or TVP) were implanted alongside LPs
7. Studies not involving humans
8. Studies reported in languages other than English

Inclusion and exclusion criteria were set to account for potential selection bias.

### 2.3 Data extraction

Two authors (N.I. and Y.I.) independently reviewed all relevant studies using predefined inclusion and exclusion criteria to identify eligible studies. Any disagreements were resolved through re‐evaluation and discussion with a third author (S.M.) who assisted with the data extraction. The extracted data included patient characteristics (age, sex, body mass index, left ventricular ejection fraction, and comorbidities), reasons for CIED removal (isolated pocket infection and systemic infection), infectious organisms, type of CIED removed, timing of CIED removal and LP implantation, study setting, number of patients, follow‐up duration, reinfection, and all‐cause mortality events.

### 2.4 Risk of bias and quality assessment

The Methodological Index for Non‐Randomized Studies (MINORS) criteria were used to assess the quality of each included study.^17^ This tool consists of a series of questions (12 and eight items for comparative and non‐comparative studies, respectively), with each item scored 0, 1, or 2. The maximum score was 24 for comparative studies and 16 for non‐comparative studies, and studies were rated as good (21–24/13–16), fair (17–20/9–12), or poor (≤16/≤8) based on their scores. Quality assessments were independently conducted by two authors (N.I. and Y.I.), and consensus was reached before proceeding to the next steps. Discrepancies were resolved through discussion with a third author (S.M.).

### 2.5 Primary and secondary outcomes

From the 16 included studies, the primary outcome was defined as the overall event incidence of all‐cause mortality and reinfection following successful CIED removal and LP implantation. The occurrence of these events was evaluated. Secondary outcomes included the incidence rates of all‐cause mortality and reinfection individually. Reinfection was defined as detection of the same pathogen that caused the initial CIED infection after successful LP implantation and discharge in the absence of other infectious sources.

### 2.6 Stratification by timing of LP implantation

To compare outcomes based on the timing of LP implantation and CIED extraction, the following two groups were defined and stratified:

1) LP implantation before or simultaneously with extraction group: Cases in which LP implantation was performed before or at the same time as the extraction of the infected CIEDs.

2) Post‐extraction LP implantation group: Cases in which LP implantation was performed in a staged manner after a defined interval following the extraction of the infected CIEDs.

From each primary study, the total number of cases and the number of events meeting these conditions were extracted. Weighted estimated proportions were then calculated, and a meta‐analysis was performed using a random‐effects model. The results were presented in a Forest plot.

### 2.7 Statistical analysis

For this analysis, which included case series and non‐comparative studies, we used the previously reported meta packages (Metaprop) to calculate the pooled incidence of complications (reinfection and all‐cause mortality).^18^, ^19^ This command utilizes the Freedman‐Tukey double arcsine transformation to incorporate studies with incidence rates of 0% or 100%. A random‐effects model (DerSimonian and Laird method) was applied to pool transformed estimates, considering the heterogeneity across studies.

Inter‐study heterogeneity was assessed using the *I* ^2^ statistic. To address the zero‐event problem, a correction of 0.5 was added to the frequency of outcomes, stabilizing the estimates by preventing zero in the denominator and minimizing excessive variation in confidence intervals and estimates. In cases of very small sample sizes, an additional correction of 1 was applied to reduce potential bias.^20^, ^21^

The pooled proportions estimated using the random‐effects model were compared between the simultaneous and post‐extraction implantations using the z‐test. A previous review recommended evaluating infection rates 1 year after CIED placement.^4^ However, since some studies included in this meta‐analysis had follow‐up periods of less than 1 year, we conducted a subgroup analysis based on follow‐up duration. Additionally, a sensitivity analysis was conducted to exclude studies that did not distinguish data from non‐infectious causes (e.g., device failure and cardiac function issues). The pooled results were illustrated using forest plots and publication bias was assessed using a funnel plot. Furthermore, Egger's linear regression test was performed to assess the symmetry of the funnel plot. Continuous variables were reported as mean ± standard deviation (SD) and categorical variables as totals and percentages.

Statistical significance was defined as a two‐sided p‐value <0.05, and all statistical analyses were conducted using R statistical software version 4.3.2 (R Foundation for Statistical Computing, United States).

## 3 RESULTS

### 3.1 Literature search and quality assessment of included studies

**FIGURE 1:**
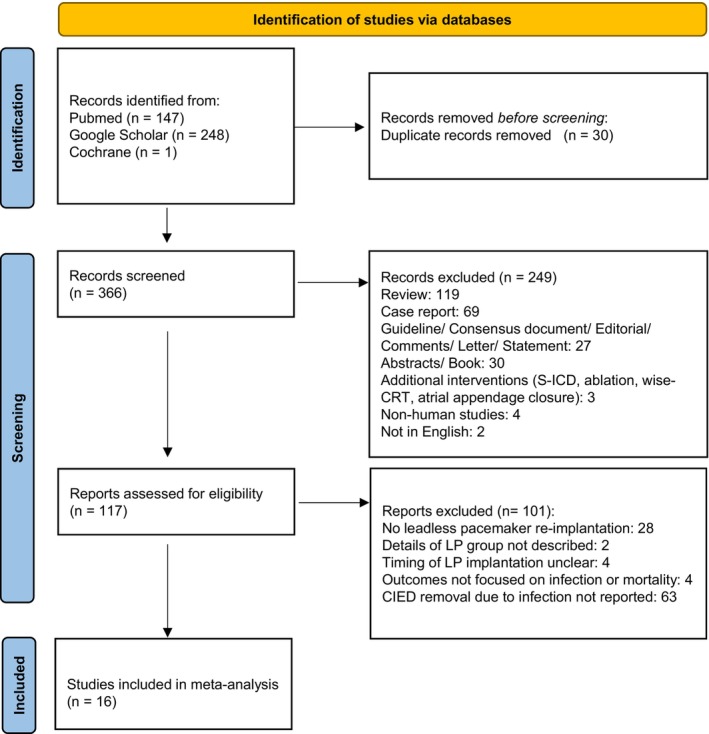
PRISMA flow diagram illustrating the search strategy and selection process for included studies.

**FIGURE 2:**
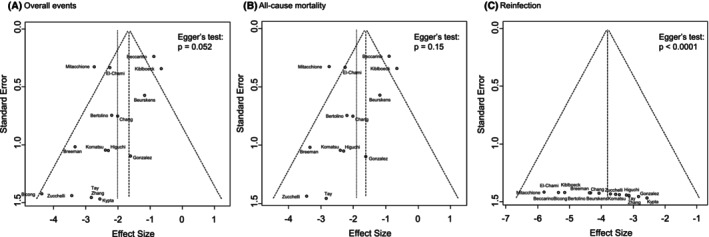
Funnel plot and Egger's linear regression test results for various outcomes. No asymmetry was observed for overall events (A) or all‐cause mortality (B), whereas publication bias was suggested for reinfection (C).

Of the 396 studies screened, 16 were included in this analysis.^22^, ^23^, ^24^, ^25^, ^26^, ^27^, ^28^, ^29^, ^30^, ^31^, ^32^, ^33^, ^34^, ^35^, ^36^, ^37^ The details are provided in Figure 1 and summarized in Table 1. Among these studies, one (6.2%) was prospective, seven (43.8%) were retrospective, and four (25%) were case series. Regarding study size, nine studies (56.2%) included fewer than 20 cases, whereas seven studies (43.8%) included 20 or more cases. The interval between CIED removal and LP implantation ranged from 2 days before removal (median) to 208 months after removal. The quality assessment, using the MINORS criteria, rated two studies (12.5%) as GOOD, 12 studies (75%) as FAIR, and two studies (12.5%) as POOR (Table 1; Table S2). A funnel plot was used to assess publication bias across all included studies (Figure 2(A–C)). Overall, most studies were symmetrically distributed around the pooled effect estimate, although some were asymmetrically positioned on the left side of the plot.

**TABLE 1:** Summary of studies included in the meta‐analysis.

Author (year)	Study setting	Number of study patients (only infection)	LP implantation before or simultaneously with extraction (only infection)	Post‐extraction LP implantation (only infection)	Duration between extraction and LP implantation median [IQR]		Follow‐up duration median [IQR] or mean ± SD
Komatsu et al. (2024)	Retrospective, single‐center cohort study	12	0	5	10 [9–13] days		329 [251–368] days
Kiblboeck et al. (2024)	Prospective, single‐center cohort study	48 (38)	35	13	Before: 2 [1–2] days After: 6 [3–11] days		15 [12–41] months
Breeman et al. (2023)	Retrospective, single‐center cohort study	29	9	20	8 [1–21] days		32 [13–66] months
Bertolino et al. (2023)	Retrospective, single‐center cohort study	49 (20)	14	4	Within 48 h post‐extraction (Two patients) After 48 h post‐extraction (Two patients)		403 [34–1666] days
Beccarino et al. (2023)	Single‐center cohort study	86	86	0	0		163 [57–403] days
Mitacchione et al. (2023)	Retrospective, multi‐center cohort study	1179 (165)^a^	0	184^c^ (165)	12 [9–15] days		33 [24–47] months
Bicong et al. (2022)	Retrospective, single‐center cohort study	39	9	30	4 [0–10] days		24.8 ± 14.7 months
Tay et al. (2022)	Single‐center case series	10 (8)	1	7	Average: 10 days		12 month
Higuchi et al. (2021)	Single‐center case series	11	1	10	14 [8–21] days		17.2 ± 6.5 months
Zhang et al. (2021)	Single‐center case series	8	8	0	0		1–10 months
Chang et al. (2020)	Retrospective, single‐center cohort study	17	17	0	0		143 [57–181] days
Beurskens et al. (2019)	Single‐center case series	17	0	17	Within 7 days post‐extraction (six patients) After 7 days post‐extraction (eleven patients)		16 ± 12 months
El‐Chami et al. (2019)	Multicenter‐cohort study	105	39	66	6 [0–10] days		8.5 ± 7.1 months
Zucchelli et al. (2019)	Single‐center cohort study	83 (15)^b^	2 (0)	21 (8)	0 days to 208 months		18 [1–24] months
Gonzalez et al. (2019)	Retrospective, single‐center cohort study	9 (6)	6	0	0		90 days
Kypta et al. (2016)	Single‐center cohort study	6	2	4	2 h to 2 days		6 months
Author (year)	Number of overall outcomes^d^	Number of all‐cause mortality	Number of reinfection	Isolated pocket infection^e^	Systemic infection^f^	Non‐infection	MINORS
Komatsu et al. (2024)	1	1	0	8	4	0	FAIR
Kiblboeck et al. (2024)	13	13	0	28	10	10	FAIR
Breeman et al. (2023)	1	1	0	21	8	0	FAIR
Bertolino et al. (2023)	4	2	0	3	17	29	FAIR
Beccarino et al. (2023)	25	25	0	21	65	0	FAIR
Mitacchione et al. (2023)	10	10	0	No repo	165^g^	19	GOOD
Bicong et al. (2022)	0	No repo	0	17	22	0	FAIR
Tay et al. (2022)	0	0	0	5	3	2	POOR
Higuchi et al. (2021)	1	1	0	5	6	0	FAIR
Zhang et al. (2021)	0	No repo	0	8	0	0	FAIR
Chang et al. (2020)	2	2	0	7	10	0	FAIR
Beurskens et al. (2019)	4	4	0	7	10	0	GOOD
El‐Chami et al. (2019)	12	10	0	No repo	No repo	No repo	FAIR
Zucchelli et al. (2019)	0	0	0	11	4	8	FAIR
Gonzalez et al. (2019)	1	1	0	5	1	3	POOR
Kypta et al. (2016)	0	No repo	0	3	3	0	FAIR

Abbreviations: IQR, interquartile range; LPs, leadless pacemakers; MINORS, the methodological index for non‐randomized studies; SD, standard deviation.

^a^ The overall number of transvenous lead extractions is 184.

^b^ The overall number of transvenous lead extractions is 23.

^c^ The timing of LP implantation for noninfective CIED‐related causes was defined according to each center's clinical practice (same stage or postponed).

^d^ Reinfection or all‐cause mortality.

^e^ Includes perforation and erosion.

^f^ Includes bacteremia, infective endocarditis, and lead infection.

^g^ The distinction between local and systemic infection is unknown.

To test for asymmetry, Egger's regression test was performed. The results showed no evidence of asymmetry in the primary outcome (intercept = −1.45, 95% CI: −2.92–0.013, *p* = .052). Similarly, no asymmetry was observed in the secondary outcome of all‐cause mortality (intercept = −1.4036, 95% CI: −3.45–0.65, *p* = .15). However, for reinfection, statistically significant asymmetry was detected, strongly suggesting the presence of publication bias (intercept = 54.2, 95% CI: 40.5–67.9, *p* < 0.0001).

### 3.2 Patient characteristics

Among all studies, 653 patients were included (Table 2). The mean age was 76.9 years (95% CI: 73.7–80.2 years), and 69.1% (95% CI: 58.4%–78.0%) were men. The mean left ventricular ejection fraction (LVEF) was 55.7% (95% CI: 52.0%–59.4%). Hypertension was present in 61.5% (95% CI: 46.2%–74.8%), diabetes in 31.6% (95% CI: 25.0%–39.0%), and chronic kidney disease (25.5%; 95% CI: 15.5%–38.9%).

**TABLE 2:** Summary of patient characteristics.

	No. of studies (*n* = 16) (No. of patients)	Summary estimate (95% confidence interval)
Study characteristics		
Prospective	1	6.2%
Retrospective	7	43.8%
Case‐series	4	25%
Not reported	4	25%
Number of patients enrolled^a^		
Studies enrolling <20 patients	9	56.2%
Studies enrolling ≥20 patients	7	43.8%
Patient demographics		
Age, year, pooled mean	16 (653 patients)	76.9 (73.7‐80.2)
Men	16 (436 patients)	69.1% (58.4%‐78.0%)
BMI, kg/m^2^	9 (393 patients)	25.1 (23.5‐26.6)
LVEF, %	8 (396 patients)	55.7 (52.0‐59.4)
Comorbidities		
Hypertension	14 (370 patients)	61.5% (46.2%‐74.8%)
Diabetes mellitus	16 (198 patients)	31.6% (25.0%‐39.0%)
Chronic kidney disease	14 (110 patients)	25.5% (15.5%‐38.9%)
COPD	9 (42 patients)	14.0% (10.5%‐18.4%)
Sinus node dysfunction	15 (104 patients)	16.8% (12.3%‐22.5%)
Atrioventricular block^b^	15 (236 patients)	40.1% (29.1%‐52.1%)
AF brady^c^	15 (191 patients)	30.0% (20.7%‐41.3%)
Reasons for CIED extraction		
Isolated pocket infection^d^	14 (149 patients)	46.7% (32.7%‐61.2%)
Systemic infection^e^	15 (328 patients)	46.3% (29.5%‐64.0%)
Baterial strain		
Staphylococcus aureus^f^	9 (89 patients)	33.3% (27.8%‐39.2%)
CNS^g^	7 (48 patients)	25.3% (17.0%‐35.8%)
Other GPC^h^	8 (47 patients)	19.1% (12.7%‐27.7%)
Extracted devices		
Single chamber‐PM^i^	15 (218 patients)	27.7% (16.3%‐43.0%)
Dual chamber‐PM	12 (292 patients)	60.6 (47.0%‐72.7%)
Single ICD^j^	15 (15 patients)	3.93% (2.53%‐6.04%)
Dual ICD	11 (9 patients)	4.18% (2.36%‐7.32%)
CRT‐P	15 (24 patients)	5.97% (4.16%‐8.50%)
CRT‐D	15 (38 patients)	7.48% (4.05%‐13.4%)
Device upgrade or additional treatment from LPs		
Dual chamber‐PM	2 (7 patients)	3.18% (0.45%‐19.1%)
ICDs	2 (5 patients)	13.6% (5.77%‐28.8%)
CRT‐P	2 (2 patients)	1.72% (0.43%‐6.64%)
CRT‐D	2 (6 patients)	2.93% (0.51%‐15.1%)
Change of LPs	2 (3 patients)	3.16% (1.02%‐9.35%)
Details unknown	1 (2 patients)	1.90% (0.48%‐7.29%)
Study design of timing with LP implantation		
Implantation before or simultaneously with extraction only	4	25%
Post‐extraction implantation only	4	25%
Both	8	50%
Quality assessment		
Good	2	12.5%
Fair	12	75%
Poor	2	12.5%

Abbreviations: AF brady, atrial fibrillation with bradycardia; BMI, body mass index; CIEDs, cardiac implantable electronic devices; CNS, coagulase‐negative staphylococci; COPD, chronic obstructive pulmonary disease; CRT‐D, cardiac resynchronization therapy‐defibrillator; CRT‐P, cardiac resynchronization therapy‐pacemaker; GPC, gram‐positive cocci; ICD, implantable cardioverter‐defibrillator; LP, leadless pacemaker; LVEF, left ventricular ejection fraction; PM, pacemaker.

^a^ Number of study patients due to infection.

^b^ No distinction of atrioventricular block degree.

^c^ Includes atrioventricular block complications.

^d^ Includes perforation and erosion.

^e^ Includes bacteremia, infective endocarditis, and lead infection.

^f^ Includes methicillin‐sensitive Staphylococcus aureus and methicillin‐resistant Staphylococcus aureus.

^g^ Includes methicillin‐sensitive Staphylococcus epidermidis, methicillin‐resistant Staphylococcus epidermidis, and coagulase‐negative staphylococci.

^h^ Includes Enterococcus and Streptococcus.

^i^ Includes pacemakers that do not distinguish between single and dual chambers.

^j^ Includes implantable cardioverter‐defibrillators that do not distinguish between single and dual chambers.

Reasons for CIED removal were nearly evenly split between isolated pocket infections (46.7%, 95% CI: 32.7%–61.2%) and systemic infections (46.3%, 95% CI: 29.5%–64.0%), including endocarditis, bacteremia, and lead infections. The most common causative organism was *Staphylococcus aureus* (33.3%, 95% CI: 27.8%–39.2%), followed by coagulase‐negative *staphylococci* (CNS) at 25.3% (95% CI: 17.0%–35.8%).

The most frequently removed device type was the dual‐chamber pacemaker, accounting for 60.6% (95% CI: 47.0%–72.7%), followed by single‐chamber pacemakers at 27.7% (95% CI: 16.3%–43.0%).

### 3.3 Analysis of primary and secondary outcomes

**FIGURE 3:**
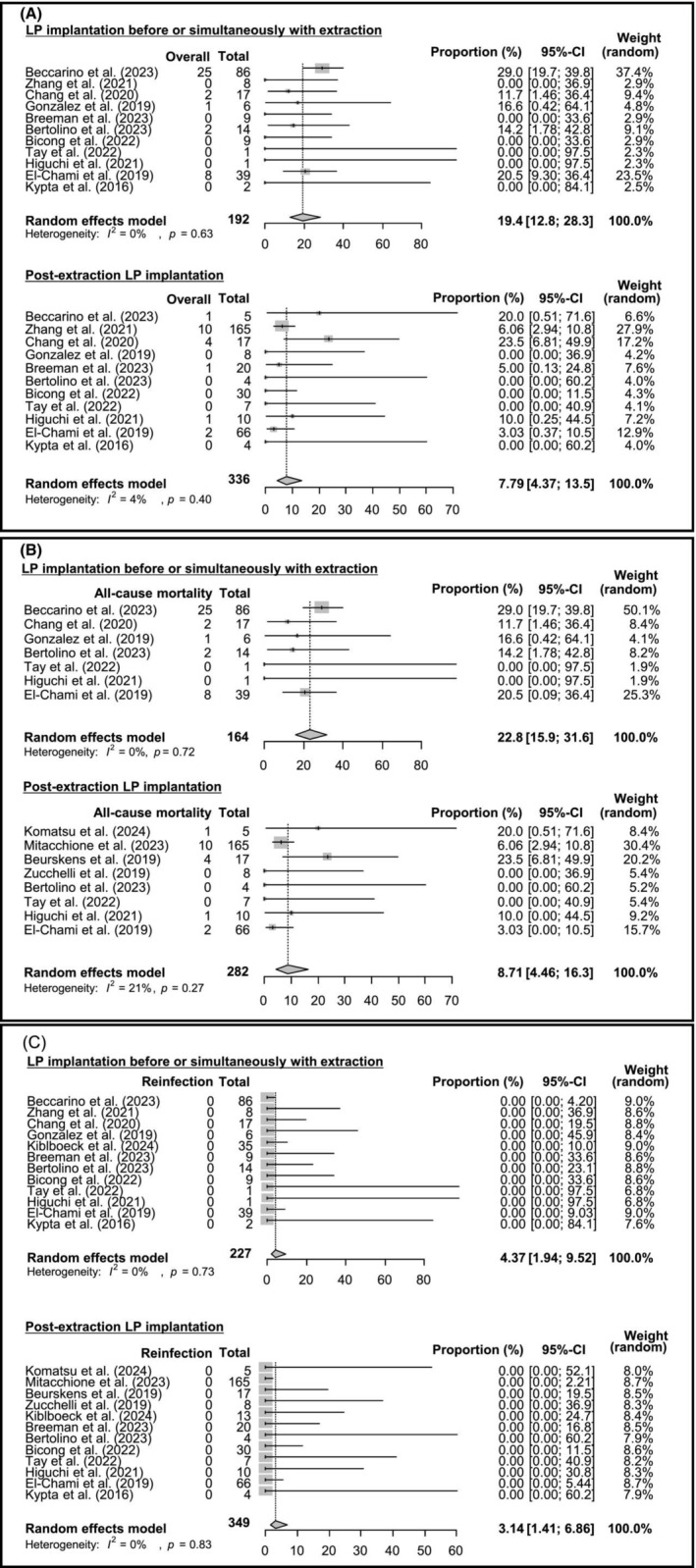
Forest plot for primary and secondary outcomes. Forest plot displaying the estimated proportions and 95% CIs for each adverse event stratified by LP implantation timing. Pooled proportions were estimated using a random‐effects model for LP implantation before or simultaneously with the extraction and Post‐extraction LP implantation groups.

The mean follow‐up period across studies was 14.0 ± 9.3 months. The pooled proportion of LP implantation for the primary outcome in the group where LP implantation was performed before or simultaneously with extraction was 19.4% (95% CI: 12.8%–28.3%), with no heterogeneity (*I* ^2^ = 0%, *p* = .63; Figure 3(A)). For the post‐extraction LP implantation group, the proportion was 7.79% (95% CI: 4.37%–13.5%), also showing no heterogeneity (*I* ^2^ = 4%, *p* = .40; Figure 3(A)).

The pooled proportion of LP implantation for the all‐cause mortality in the group where LP implantation was performed before or simultaneously with extraction was 22.8% (95% CI: 15.9%–31.6%), with no heterogeneity (*I* ^2^ = 0%, *p* = .72; Figure 3(B)), and for the post‐extraction LP implantation group, it was 8.71% (95% CI: 4.46%–16.3%, *I* ^2^ = 21%, *p* = .27; Figure 3(B)).

Reinfection events were not reported in any of these studies (Figure 3(C)).

**FIGURE 4:**
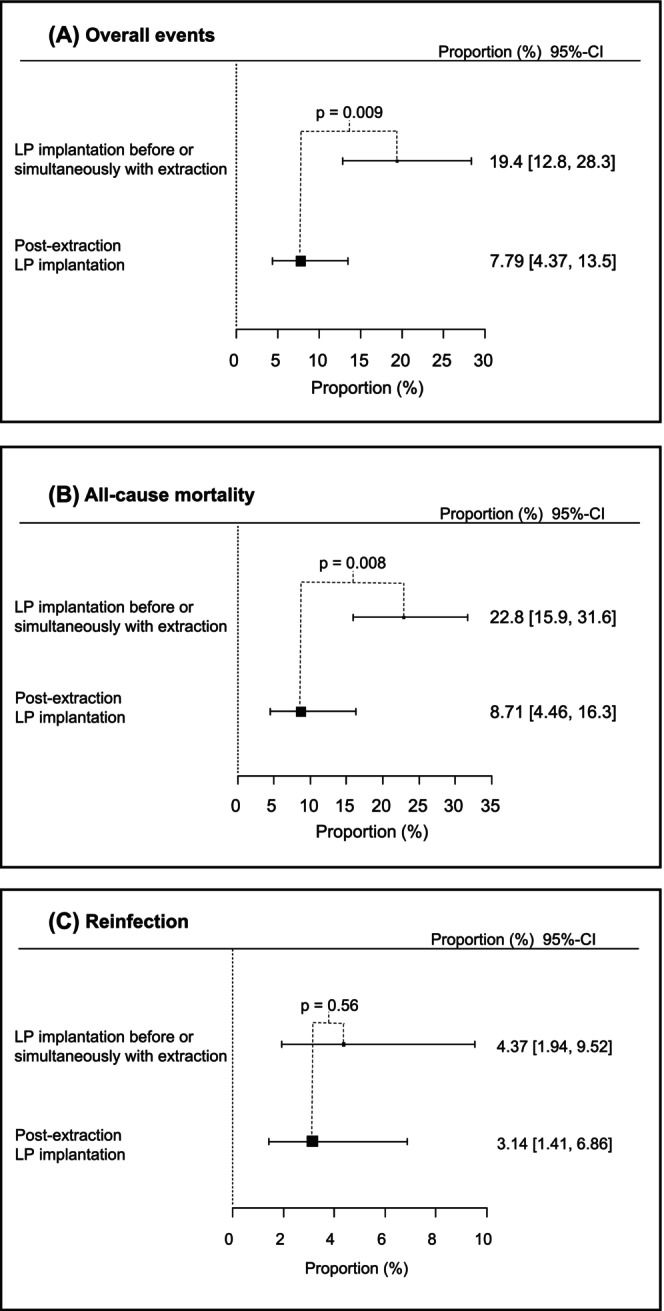
Comparison of pooled proportions for each event between the LP implantation before or simultaneously with extraction and Post‐extraction LP implantation groups. A significant difference was observed between the two groups in terms of Overall Events (*p* = .009) and All‐Cause Mortality (*p* = .008).

A comparison of the estimated proportions of outcomes between the LP implantation before or simultaneously with the extraction and post‐extraction implantation groups revealed an overall outcome (*p* = .009), all‐cause mortality (*p* = .008), and reinfection (*p* = .56; Figure 4(A–C)).

### 3.4 Subgroup analysis of all‐cause mortality by follow‐up period

Seven studies had a median observation period of less than 1 year, while nine studies had a period of 1 year or more. To evaluate trends in all‐cause mortality based on differences in the follow‐up duration, we conducted a subgroup analysis. The results are presented in Figure S1.

### 3.5 Sensitivity analysis

A sensitivity analysis was conducted, excluding seven studies that could not differentiate between infectious and non‐infectious causes due to data extraction limitations, leaving nine studies.

For overall outcomes, the pooled proportion in the simultaneous implantation group was 17.0% (95% CI: 8.32%–31.8%), with *I* ^2^ = 11% (*p* = .34), and 11.8% (95% CI: 5.24%–24.7%), with *I* ^2^ = 7% (*p* = .37) in the post‐extraction group. Detailed results of the secondary outcomes are presented in Figure S2.

## 4 DISCUSSION

In this meta‐analysis, we demonstrated that performing LP implantation simultaneously with CIED removal due to infection was associated with an increased risk of all‐cause mortality. Previous studies were small sample‐sized, single‐center, or case series, and although some reviews have been reported,^1^, ^25^ no meta‐analysis to date has distinguished outcomes according to LP implantation timing.

Furthermore, the identification of a statistically significant difference in all‐cause mortality represents a novel contribution to this meta‐analysis.

New antimicrobial strategies for CIED infections, including PADIT,^38^ have been reported,^39^ yet current guidelines continue to recommend complete removal.^40^ For patients who are dependent on pacing, LPs—considered to have a lower infection risk^41^—are valuable. However, cases of LP‐related infections following de novo LP implantation have been reported,^42^, ^43^ and real‐world data on LP implantation during CIED infection remain insufficient.

In a study by Tan et al., involving 253 patients from 13 studies who underwent LP implantation after TLE (mean interval to implantation: 5.4 days), the LP‐related infection rate was 0.4% (1/253), suggesting that LP implantation post‐TLE is safe.^1^ Although there was some numerical discrepancy in reinfection due to the estimated pooled incidence rate in our meta‐analysis, no statistically significant difference was observed when comparing LP implantation timing. However, the estimated mortality rate during the follow‐up period ranged from 8.71% to 22.8%, which is relatively high. Considering that all‐cause mortality could act as a competing risk for reinfection, the reliability of the risk assessment for reinfection associated with LP implantation may be compromised.

Furthermore, although not included in our review as it was a research letter, a study by Maille et al. on Micra implantation following TLE showed a 1‐month all‐cause mortality of 6.8% in the two‐in‐one group (those who underwent simultaneous TLE and Micra implantation) and a mortality rate of 20.5% at a median follow‐up of 186 days.^10^

These findings, along with the results of this meta‐analysis, suggest the possibility of underestimating the reinfection risk due to the competing risk of all‐cause mortality.

Therefore, further large‐scale studies on LP implantation in the context of infections are warranted.

## 5 LIMITATIONS

This meta‐analysis had some limitations. First, the clinical significance of CIEDs varies depending on whether a pacemaker or ICD/CRT‐D is extracted and replaced with an LP. Although we selected all‐cause mortality as the outcome, many studies did not provide detailed causes of death, limiting our ability to specifically evaluate cardiovascular mortality.

Second, whether the infection leading to CIED extraction was an isolated pocket infection or systemically likely impacts the risk of LP‐related infections. Our analysis revealed similar proportions of infection types. Although we conducted a subgroup analysis by infection type, few studies provided detailed information on this relationship, preventing further analysis.

Finally, primary studies inadequately documented antibiotic therapy details (treatment regimen and duration) and criteria for determining LP implantation timing post‐treatment. While some studies mentioned antibiotic use, it was unclear when treatment was deemed sufficient or when the criteria for appropriate device removal and LP implantation were met. Therefore, in cases of simultaneous LP implantation and removal, infections may only have partially resolved, suggesting that simultaneous removal and LP implantation should be approached cautiously.

## 6 CONCLUSIONS

Many studies have reported that performing LP implantation simultaneously with CIED extraction due to infection is not associated with an increased risk of subsequent reinfection. However, all‐cause mortality tends to increase with simultaneous implantation, raising concerns about competing risks and highlighting the need for further research.

## FUNDING INFORMATION

This research did not receive any grants from funding agencies in public.

## CONFLICT OF INTEREST STATEMENT

The authors declare no conflicts of interest.

## ETHICS STATEMENT

In line with our institutional policy, the systematic review and meta‐analysis did not require approval from the Institutional Review Board. We ensured compliance with ethical guidelines and maintained the highest standards of integrity throughout all processes.

## Supporting information


Figure S1.

Figure S2.

Data S1.
