# Supplementary material for: Assessment of adverse events stratified by timing of leadless pacemaker implantation with cardiac implantable electronic devices extraction due to infection: A systematic review and meta‐analysis
Source: J Arrhythm. 2024 Dec 26;41(1):e13208. doi: 10.1002/joa3.13208 (PMC11730721; doi:10.1002/joa3.13208)
Supplement: Supplementary file 1 — Figure S1. [file JOA3-41-e13208-s002.pptx]

## Slide 1
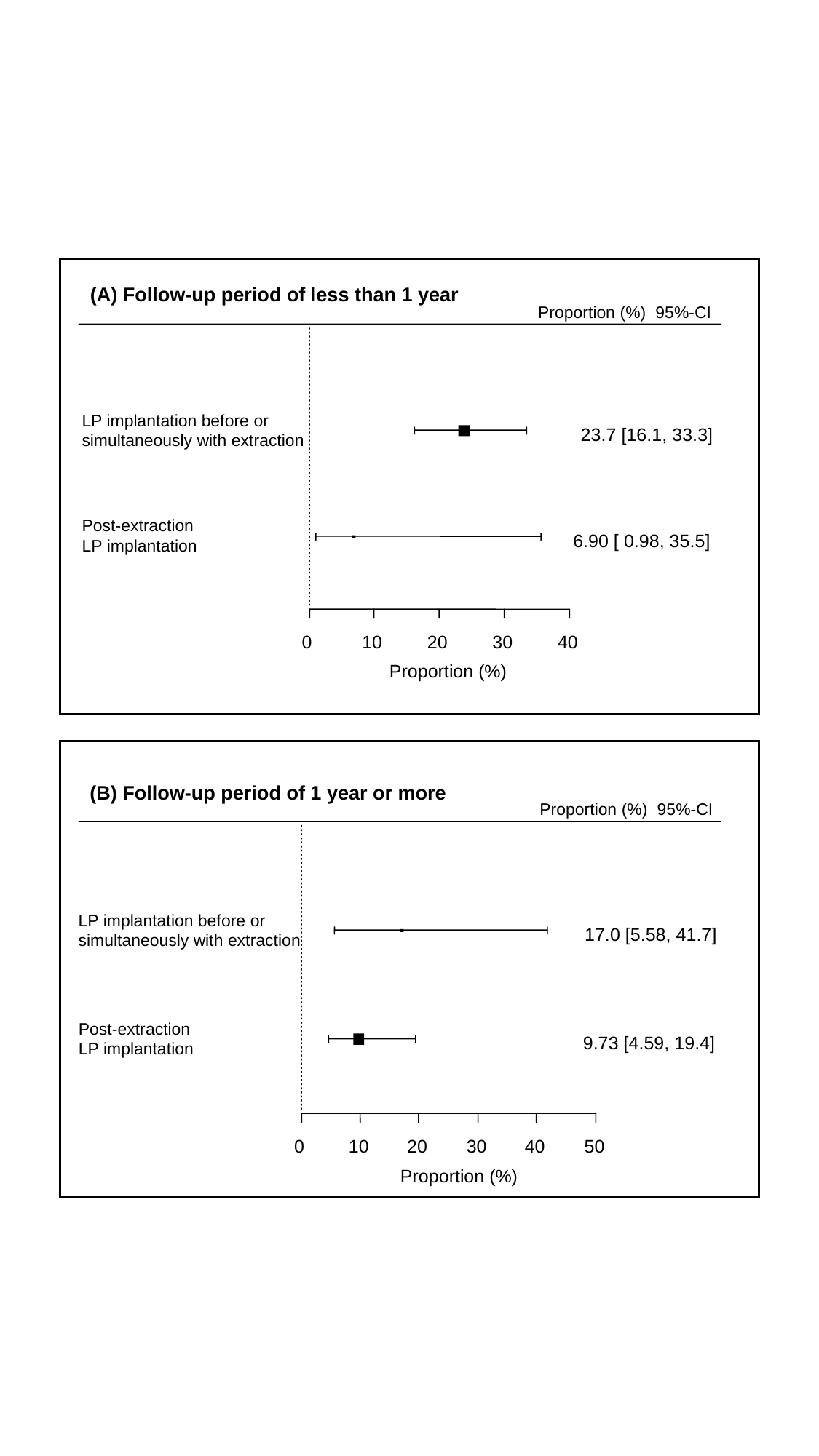

(A) Follow-up period of less than 1 year
Proportion (%) 95%-CI
LP implantation before or
simultaneously with extraction
23.7 [16.1, 33.3]
Post-extraction
LP implantation
 6.90 [ 0.98, 35.5]
0
10
20
30
40
Proportion (%)
Proportion (%) 95%-CI
(B) Follow-up period of 1 year or more
17.0 [5.58, 41.7]
 9.73 [4.59, 19.4]
0
10
20
30
40
50
Proportion (%)
LP implantation before or
simultaneously with extraction
Post-extraction
LP implantation
