# Supplementary material for: Assessment of adverse events stratified by timing of leadless pacemaker implantation with cardiac implantable electronic devices extraction due to infection: A systematic review and meta‐analysis
Source: J Arrhythm. 2024 Dec 26;41(1):e13208. doi: 10.1002/joa3.13208 (PMC11730721; doi:10.1002/joa3.13208)
Supplement: Supplementary file 2 — Figure S2. [file JOA3-41-e13208-s001.pptx]

## Slide 1
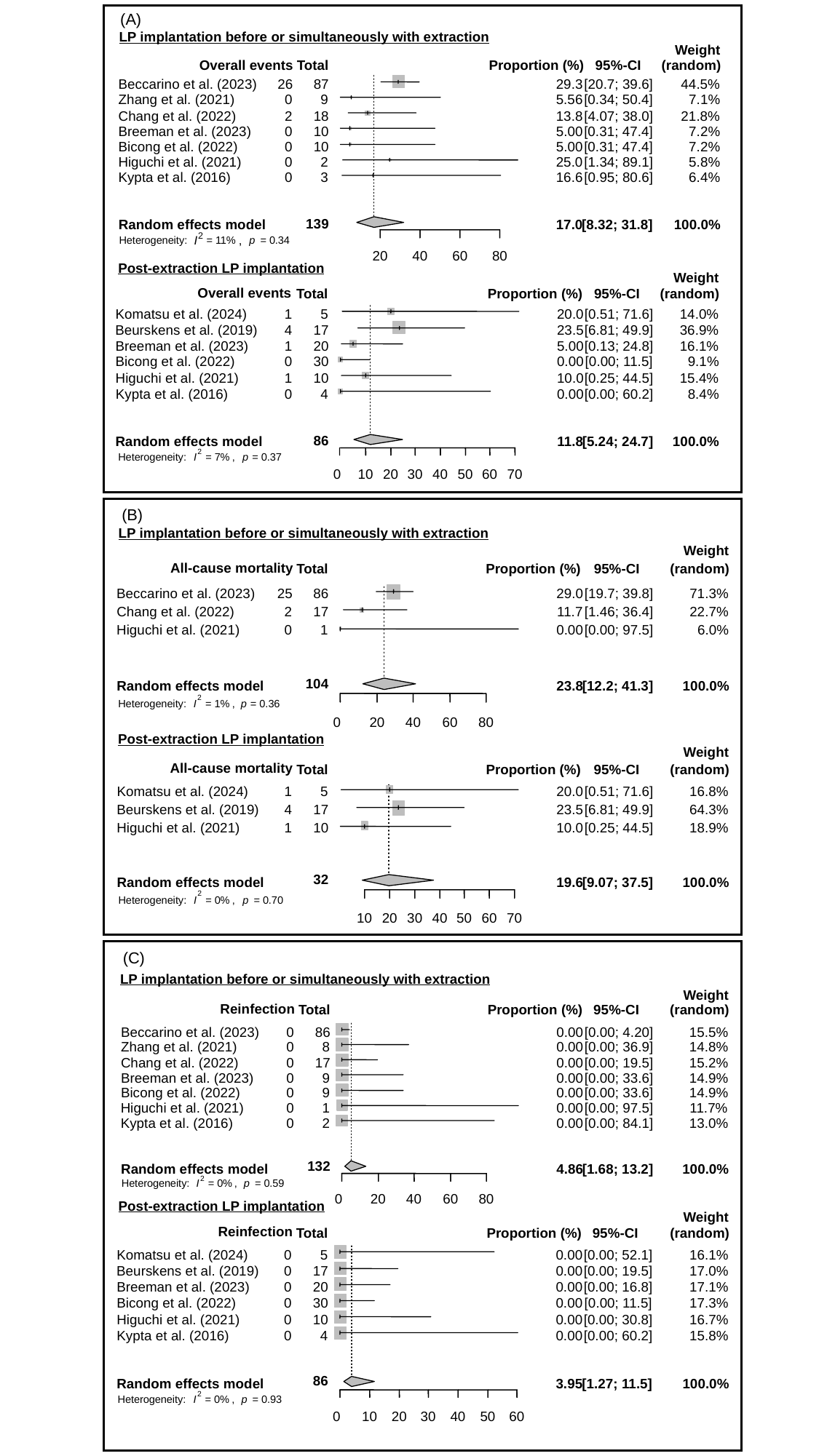

(A)
LP implantation before or simultaneously with extraction
Weight
Overall events
Total
Proportion (%)
95%-CI
(random)
Beccarino et al. (2023)
26
87
29.3
[20.7; 39.6]
44.5%
Zhang et al. (2021)
0
9
5.56
[0.34; 50.4]
7.1%
Chang et al. (2022)
2
18
13.8
[4.07; 38.0]
21.8%
Breeman et al. (2023)
0
10
5.00
[0.31; 47.4]
7.2%
Bicong et al. (2022)
0
10
5.00
[0.31; 47.4]
7.2%
Higuchi et al. (2021)
0
2
25.0
[1.34; 89.1]
5.8%
Kypta et al. (2016)
0
3
16.6
[0.95; 80.6]
6.4%
139
Random effects model
17.0
[8.32; 31.8]
100.0%
2
Heterogeneity:
I
 = 11%
,
p
 = 0.34
20
40
60
80
Post-extraction LP implantation
Weight
Overall events
Total
Proportion (%)
95%-CI
(random)
Komatsu et al. (2024)
1
5
20.0
[0.51; 71.6]
14.0%
Beurskens et al. (2019)
4
17
23.5
[6.81; 49.9]
36.9%
Breeman et al. (2023)
1
20
5.00
[0.13; 24.8]
16.1%
Bicong et al. (2022)
0
30
0.00
[0.00; 11.5]
9.1%
Higuchi et al. (2021)
1
10
10.0
[0.25; 44.5]
15.4%
Kypta et al. (2016)
0
4
0.00
[0.00; 60.2]
8.4%
86
Random effects model
11.8
[5.24; 24.7]
100.0%
2
Heterogeneity:
I
 = 7%
,
p
 = 0.37
0
10
20
30
40
50
60
70
(B)
LP implantation before or simultaneously with extraction
Weight
All-cause mortality
Total
Proportion (%)
95%-CI
(random)
Beccarino et al. (2023)
25
86
29.0
[19.7; 39.8]
71.3%
Chang et al. (2022)
2
17
11.7
[1.46; 36.4]
22.7%
Higuchi et al. (2021)
0
1
0.00
[0.00; 97.5]
6.0%
104
Random effects model
23.8
[12.2; 41.3]
100.0%
2
Heterogeneity:
I
 = 1%
,
p
 = 0.36
0
20
40
60
80
Post-extraction LP implantation
Weight
All-cause mortality
Total
Proportion (%)
95%-CI
(random)
Komatsu et al. (2024)
1
5
20.0
[0.51; 71.6]
16.8%
Beurskens et al. (2019)
4
17
23.5
[6.81; 49.9]
64.3%
Higuchi et al. (2021)
1
10
10.0
[0.25; 44.5]
18.9%
32
Random effects model
19.6
[9.07; 37.5]
100.0%
2
Heterogeneity:
I
 = 0%
,
p
 = 0.70
10
20
30
40
50
60
70
(C)
LP implantation before or simultaneously with extraction
Weight
Reinfection
Total
Proportion (%)
95%-CI
(random)
Beccarino et al. (2023)
0
86
0.00
[0.00; 4.20]
15.5%
Zhang et al. (2021)
0
8
0.00
[0.00; 36.9]
14.8%
Chang et al. (2022)
0
17
0.00
[0.00; 19.5]
15.2%
Breeman et al. (2023)
0
9
0.00
[0.00; 33.6]
14.9%
Bicong et al. (2022)
0
9
0.00
[0.00; 33.6]
14.9%
Higuchi et al. (2021)
0
1
0.00
[0.00; 97.5]
11.7%
Kypta et al. (2016)
0
2
0.00
[0.00; 84.1]
13.0%
132
Random effects model
4.86
[1.68; 13.2]
100.0%
2
Heterogeneity:
I
 = 0%
,
p
 = 0.59
0
20
40
60
80
Post-extraction LP implantation
Weight
Reinfection
Total
Proportion (%)
95%-CI
(random)
Komatsu et al. (2024)
0
5
0.00
[0.00; 52.1]
16.1%
Beurskens et al. (2019)
0
17
0.00
[0.00; 19.5]
17.0%
Breeman et al. (2023)
0
20
0.00
[0.00; 16.8]
17.1%
Bicong et al. (2022)
0
30
0.00
[0.00; 11.5]
17.3%
Higuchi et al. (2021)
0
10
0.00
[0.00; 30.8]
16.7%
Kypta et al. (2016)
0
4
0.00
[0.00; 60.2]
15.8%
86
Random effects model
3.95
[1.27; 11.5]
100.0%
2
Heterogeneity:
I
 = 0%
,
p
 = 0.93
0
10
20
30
40
50
60
